# Fitness, fatness, and academic performance in seventh-grade elementary school students

**DOI:** 10.1186/1471-2431-14-176

**Published:** 2014-07-07

**Authors:** Luís B Sardinha, Adilson Marques, Sandra Martins, António Palmeira, Cláudia Minderico

**Affiliations:** 1 Interdisciplinary Center for the Study of Human Performance, Faculty of Human Kinetics, University of Lisbon, Estrada da Costa, 1499-002 Cruz-Quebrada, Portugal; 2 Faculty of Physical Education and Sport, Lusophone University of Humanities and Technologies, Lisbon, Portugal

**Keywords:** Physical fitness, Academic performance, Weight status, School

## Abstract

**Background:**

In addition to the benefits on physical and mental health, cardiorespiratory fitness has shown to have positive effects on cognition. This study aimed to investigate the relationship between cardiorespiratory fitness and body weight status on academic performance among seventh-grade students.

**Methods:**

Participants included 1531 grade 7 students (787 male, 744 female), ranging in age from 12 to 14 years (*M*_age_ = 12.3 ± 0.60), from 3 different cohorts. Academic performance was measured using the marks students had, at the end of their academic year, in mathematics, language (Portuguese), foreign language (English), and sciences. To assess cardiorespiratory fitness the Progressive Aerobic Cardiovascular Endurance Run, from Fitnessgram, was used as the test battery. The relationship between academic achievement and the independent and combined association of cardiorespiratory fitness/weight status was analysed, using multinomial logistic regression.

**Results:**

Cardiorespiratory fitness and weight status were independently related with academic achievement. Fit students, compared with unfit students had significantly higher odds for having high academic achievement (OR = 2.29, 95% CI: 1.48-3.55, *p* < 0.001). Likewise, having a normal weight status was also related with high academic achievement (OR = 3.65, 95% CI: 1.82-7.34, *p* < 0.001).

**Conclusions:**

Cardiorespiratory fitness and weight status were independently and combined related to academic achievement in seventh-grade students independent of the different cohorts, providing further support that aerobically fit and normal weight students are more likely to have better performance at school regardless of the year that they were born.

## Background

Physical fitness is associated with a variety of health benefits in young people and adults. Low cardiorespiratory fitness, as part of the general health-related fitness of children and adolescents, has been associated with a cluster of cardiovascular disease (CVD) risk factors [1], independent of fatness and physical activity [2], and it is well recognized as a relevant marker of cardiovascular health [3]. Low cardiorespiratory fitness is also related to obesity [4], and changes in cardiorespiratory fitness are a significant predictor of changes in fatness that occur from childhood to adolescence, even after controlling for confounding factors such as physical activity, gender, and maturity [5]. Cardiorespiratory fitness may also improve other biological outcomes such as bone mineral density [6], arterial stiffness [7] and mental health outcomes [6].

Additionally, cardiorespiratory fitness has been shown to have positive effects on cognition. The evidence is strengthened by findings from studies that report a positive relationship between cardiorespiratory fitness and academic performance among children and adolescents from elementary up to secondary school [8-12]. Exercise and physical activity have the potential to improve or maintain cardiorespiratory fitness. On the other hand, cardiorespiratory fitness affects brain plasticity [13], and it is associated with cognitive health, better cognitive abilities, larger brain structures, elevated brain function [14-16], and improved memory [17,18] along with neurocognitive functions and cognitive control [19]. Improving neurocognitive functions and the brain plasticity may result in better academic performance, as has been demonstrated in previous studies [11].

The evidence about how weight status might affect students’ school outcome is not conclusive. Some studies have not established a clear relationship between weight status and academic performance [9,20-22], while others have shown that overweight status and obesity are inversely associated with academic performance [23-26]. This controversial relationship needs to be further addressed.

There is evidence from analyses of economic outcomes that the quality of education, measured on an outcome basis of students’ cognitive skills, has a great effect on the economy [27]. If weight status and cardiorespiratory fitness are related with students’ academic achievement, policy-makers and society should recognize its importance in order to contribute to better health, education and consequently economic development. However, the importance of health-related fitness directly or indirectly in economics has been neglected by health economists [28,29].

Despite the fact that several studies have found relationships between cardiorespiratory fitness, weight status and academic performance [9,10,22,26], most of these studies did not take into consideration their dynamic changes over time in different cohorts, and the possible relationship between academic achievement and the combined association of cardiorespiratory fitness/weight status. These types of studies are important because they allow establishing a better outcome than studies with one cohort sample. Therefore, the aim of this study was to investigate the relationship between cardiorespiratory fitness, weight status and academic performance among seventh-grade students, using three cohort samples of children and adolescents.

## Methods

### Study design and participants

This study used data from the Physical Activity and Family-based Intervention in Paediatric Obesity Prevention in the School Setting (PESSOA Project). This project was applied to grade 5, 6 and 7 students from fourteen Portuguese public schools, in the Oeiras Municipality, between 2009 and 2011, and involved 4468 children and adolescents. The PESSOA program is a school-based cluster randomized controlled trial that addresses mediator variables, such as personal and social factors, and physical and social environmental factors within an ecological model that are related to and, influence physical activity. Schools were randomly allocated to one of three different groups: the first (control) group was intervened with a standard protocol with general information regarding eating and physical activity behaviours; the second group (intervention 1), besides the standard counselling, was provided a 90 min additional weekly session of physical activity; the third group (intervention 2), in addition to the standard counselling was provided a 90 min additional weekly session with health and weight educational program and physical activity, implementing principles (consistent with the tenets of the self-determination theory) and basic knowledge within the components of physical activity, eating behaviour and well-being designed to influence healthier choices.

The study received approval from the Scientific Committee of the Faculty of Human Kinetics at the University of Lisbon, the Portuguese Minister of Education, and the principals of each of the fourteen schools surveyed. The study was conducted according to ethical standards in sport and exercise science research [30]. Data were collected in the school setting after an agreement of participation of all the schools. Participants were informed about the objectives of the study and informed written consent was obtained from them and from their legal guardians.

Participation was voluntary. All healthy students that attended the physical education classes were considered eligible to participate. For the purpose the results presented in this study included 1531 grade 7 students (787 male, 744 female), ranging in age from 12 to 14 years (*M*_age_ = 12.3 ± 0.60), from 3 different cohorts. The first cohort started the study in grade 5 and was followed to grade 7. The second cohort started in grade 6 and was followed until grade 8. Finally, the last cohort started in grade 7 and finished the study in grade 9.

### Measures and procedures

Academic performance was assessed using the marks students had, at the end of their academic year, in mathematics, language (Portuguese), foreign language (English), and sciences. These marks were provided by the administrative services of each school at the end of the school year.

In Portuguese elementary schools, student marks range from 1 to 5 (1 = very poor, 2 = poor, 3 = average, 4 = good, and 5 = very good). An index of academic achievement was computed using the sum of the original marks of the four disciplines, ranging from 4 to 20. For data analysis students were grouped into low achievement (if the sum of marks of the four disciplines was between 4 and 11), average achievement (if the sum of marks of the four disciplines was between 12 and 15), and high academic achievement (if the sum of marks of the four disciplines was between 16 and 20).

To assess cardiorespiratory fitness the Progressive Aerobic Cardiovascular Endurance Run (PACER), from the Fitnessgram test battery, was used. The PACER is an incremental running test that uses a 20 metre shuttle run which progressively increases in difficulty. Participants were classified as fit and unfit according to the Fitnessgram cut points for cardiorespiratory fitness. The classification was based on gender- and age-related criterion-referenced standards. The standards are related to minimum levels of fitness that prevent diseases from a sedentary lifestyle [31].

To assess the weight status, participants were weighed to the nearest 0.1 kg wearing minimal clothes, and without shoes, and height was measured to the nearest 0.1 cm. BMI was obtained using the Quetelet index [weight (kg)/height (m)^2^]. Participants were classified into normal weight and overweight or obese, according to the gender- and age-related criterion-referenced standards by the International Obesity Task Force (IOTF) [32].

Cardiorespiratory fitness and weight status data were collected during physical education classes over a period of one week. To ensure accurate completion of Fitnessgram administration, researchers and teachers supervised the entire data collection process.

### Data analysis

Descriptive statistics were performed to characterize the sample. Bivariate relationship between academic performance and gender, and weight status and cardiorespiratory fitness were tested by the chi-square test. Multinomial logistic regression analysis was used to study the relationship between cardiorespiratory fitness, weight status and academic achievement. In the multinomial logistic regression model for academic achievement we considered three groups: (i) students with low achievement, as a reference category, (ii) students with average academic achievement, and (iii) students with high achievement. Unadjusted and adjusted odds ratio (OR) with 95% confidence intervals (CI) were calculated. Adjustments were performed by controlling for gender, weight status, cardiorespiratory fitness, and different cohorts. The OR was calculated against the reference categories of male, obese weight status, and unfit cardiorespiratory fitness. The relationship between academic achievement and the combined association of cardiorespiratory fitness/weight status was also analysed, using multinomial logistic regression. The models were adjusted for gender and cohorts. For the association of cardiorespiratory fitness/weight status, four groups were created: (i) cardiorespiratory fit/normal weight students, (ii) cardiorespiratory fit/overweight or obese students, (iii) cardiorespiratory unfit/normal weight students, and (iv) cardiorespiratory unfit/overweight or obese students. Data analysis was performed using IBM SPSS Statistics version 20 (SPSS, Chicago, IL, USA). For all tests statistical significance was set at *p* < 0.05.

## Results

The general sample’s characteristics are presented in Table 1. For weight status, the proportion of the participants who were normal weight was 69.9% and 30.3% were overweight or obese. Almost three-fourths (74.8%) of all participants were in the fit category for cardiorespiratory fitness. Overall, the majority of students passed (>2) in mathematics, language, foreign language and science, achieving an average academic performance. For the overall academic achievement 76.4% reached the low and average level while 23.6% achieved the high level.

**Table 1:** Characteristics of elementary school students from grade 7 by different cohorts

	**Cohort 1**	**Cohort 2**	**Cohort 3**	**Total**
	**n (%)**	**n (%)**	**n (%)**	**n (%)**
Gender				
Male	184 (51.3)	215 (50.7)	388 (51.9)	787 (51.4)
Female	175 (48.7)	209 (49.3)	360 (48.1)	744 (48.6)
Weight status				
Obesity	24 (7.1)	29 (7.1)	57 (7.6)	110 (7.4)
Overweight	71 (21.0)	101 (24.6)	164 (22.3)	336 (22.7)
Normal weight	243 (67.7)	280 (68.3)	513 (69.9)	1036 (69.9)
Cardiorespiratory fitness				
Unfit	101 (28.1)	119 (28.1)	166 (22.2)	386 (19.9)
Fit	258 (71.9)	305 (71.9)	582 (77.8)	1145 (74.8)
Cardiorespiratory fitness/weight status				
Unfit/overweight	46 (12.7)	56 (13.2)	71 (9.5)	173 (11.3)
Unfit/normal weight	56 (15.7)	60 (14.2)	94 (12.5)	210 (13.7)
Fit/overweight	55 (15.4)	78 (18.5)	154 (20.6)	288 (18.8)
Fit/normal weight	202 (56.2)	229 (54.1)	405 (57.4)	860 (56.2)
Mathematics				
Low achievement	70 (19.5)	113 (26.7)	187 (25.0)	370 (24.2)
Average achievement	172 (47.9)	210 (49.5)	289 (38.6)	671 (43.8)
High achievement	117 (32.6)	101 (23.8)	272 (36.4)	490 (32.0)
Portuguese language				
Low achievement	40 (11.1)	52 (12.3)	115 (15.4)	207 (13.5)
Average achievement	208 (57.9)	259 (61.1)	351 (46.9)	818 (53.4)
High achievement	111 (30.9)	113 (26.7)	282 (37.7)	506 (33.1)
Foreign language (English)				
Low achievement	54 (15.0)	76 (17.9)	118 (15.8)	248 (16.2)
Average achievement	159 (44.3)	230 (54.2)	333 (44.5)	722 (47.2)
High achievement	146 (40.7)	118 (27.8)	297 (39.7)	561 (36.6)
Sciences				
Low achievement	31 (8.6)	62 (14.6)	107 (14.3)	200 (13.1)
Average achievement	183 (51.0)	247 (58.3)	339 (45.3)	769 (50.2)
High achievement	145 (40.4)	115 (27.1)	302 (40.4)	562 (36.7)
Overall academic achievement				
Low achievement	60 (16.7)	91 (21.5)	146 (19.5)	297 (19.4)
Average achievement	213 (59.3)	267 (63.0)	392 (52.4)	872 (57.0)
High achievement	86 (24.0)	66 (15.6)	210 (28.1)	362 (23.6)

Cohort 1 started the study in grade 5, and these data were collected in 2011.

Cohort 2 started the study in grade 6, and these data were collected in 2010.

Cohort 3 started the study in grade 7, and these data were collected in 2009.

For the overall sample, students’ academic achievement did not differ significantly by gender (*χ*^2^(2) = 1.040, *p* = 0.595). However, statistically significant differences were found for weight status (*χ*^2^(4) = 32.259, *p* < 0.001), and cardiorespiratory fitness (*χ*^2^(2) = 19.983, *p* < 0.001). Those who were normal weight and presented a higher cardiorespiratory fitness had better academic achievement (Table 2). Similar results were observed for each cohort.

**Table 2:** Academic achievement by selected factors (chi-square)

		**Cohort 1**				**Cohort 2**				**Cohort 3**				**Total**		
	**L**	**A**	**H**	***p***	**L**	**A**	**H**	***p***	**L**	**A**	**H**	***p***	**L**	**A**	**H**	***p***
Gender				0.141				0.796				0.193				0.595
Male	56.7	46.9	58.1		53.8	49.8	50.0		50.7	55.9	45.2		52.9	51.8	49.2	
Female	43.3	53.1	41.9		46.2	20.2	50.0		49.3	44.1	54.8		47.1	48.2	50.8	
Weight status				0.042				0.029				0.001				<0.001
Normal weight	60.7	67.1	81.5		65.9	66.2	80.0		57.0	71.0	76.7		60.4	69.5	78.4	
Overweight	26.8	19.7	17.3		21.2	28.1	15.4		29.6	21.2	19.4		26.5	23.3	18.2	
Obesity	12.5	7.5	1.2		12.9	5.8	4.6		13.4	7.8	3.9		13.1	7.2	3.4	
Cardiorespiratory fitness				0.003				0.033				0.035				<0.001
Unfit	36.7	31.5	14.0		33.0	29.6	15.2		30.1	20.7	19.5		32.3	26.0	17.4	
Fit	63.3	68.5	86.0		67.0	70.4	84.8		69.9	79.3	80.5		67.7	74.0	82.6	

L – low academic achievement; A – average academic achievement; H – high academic achievement.

Cohort 1 started the study in grade 5, and these data were collected in 2011.

Cohort 2 started the study in grade 6, and these data were collected in 2010.

Cohort 3 started the study in grade 7, and these data were collected in 2009.

Table 3 presents the results of the unadjusted and adjusted multinomial logistic regression. Being male or female was not related to academic achievement in the unadjusted analysis. However, being female was related to high academic achievement when all the variables were adjusted to the model (OR = 1.57, 95% CI: 1.09-2.26, *p* = 0.016). For weight status, overweight or normal weight students, compared with obese students, had higher OR for having an average or high academic achievement versus those who had low academic achievement. It is important to highlight that the OR of having high or average academic achievement versus low academic achievement of normal weight students was higher than overweight in both unadjusted (OR = 4.98, 95% CI: 2.53-9.81, *p <* 0.001) and adjusted analysis (OR = 3.72, 95% CI: 1.85-7.49, *p <* 0.001). Fit students, compared with unfit students, had significantly higher odds for having a high academic achievement, in both the unadjusted (OR = 2.27, 95% CI: 1.57-3.26, *p <* 0.001), and adjusted model (OR = 2.27, 95% CI: 1.46-3.52, *p <* 0.001). This means that individually or adjusted for other variables, including the effect of the cohorts, cardiorespiratory fitness is an important predictor of high academic achievement. On the other hand, average academic achievement was only related with higher cardiorespiratory fitness in the unadjusted analysis, failing to remain significant after adjusting covariates. This reinforces the importance of cardiorespiratory fitness as a predictor of high academic achievement.

**Table 3:** Multivariate multinomial logistic regression predicting average and high overall academic achievement

**Characteristic**	**Unadjusted model**		**Adjusted model**	
	**Average academic achievement**	**High academic achievement**	**Average academic achievement**	**High academic achievement**
	**OR (95% CI)**	**OR (95% CI)**	**OR (95% CI)**	**OR (95% CI)**
Gender^a^				
Female	1.04 (0.80-1.36)	1.16 (0.85-1.58)	1.12 (0.86-1.62)	1.57 (1.09-2.26)*
Weight status^b^				
Overweight	1.59 (0.98-2.59)	2.63 (1.27-5.47)**	1.49 (0.91-2.44)	2.22 (1.05-4.66)*
Normal weight	2.09 (1.34-3.25)**	4.98 (2.53-9.81)***	1.88 (1.18-2.98)**	3.72 (1.85-7.49)***
Cardiorespiratory fitness^c^				
Fit	1.36 (1.02-1.81)*	2.27 (1.57-3.26)***	1.31 (0.92-1.88)	2.27 (1.46-3.52)***

OR – Odds ratio; CI – confidence interval.

Multinomial Logistic Regression with maximum-likelihood ratio was tested for selected variables. Low academic achievement is the reference group for outcome. In the unadjusted model each variable was tested independently as a predictor of academic achievement. In the adjusted model, the effect of cohorts was controlled and all the variables were tested in the same model, controlling the effect of each other.

^a^Reference category is "male".

^b^Reference category is "obese".

^c^Reference category is "unfit".

*p <0.05, **p < 0.01, ***p < 0.001.

**Figure 1:**
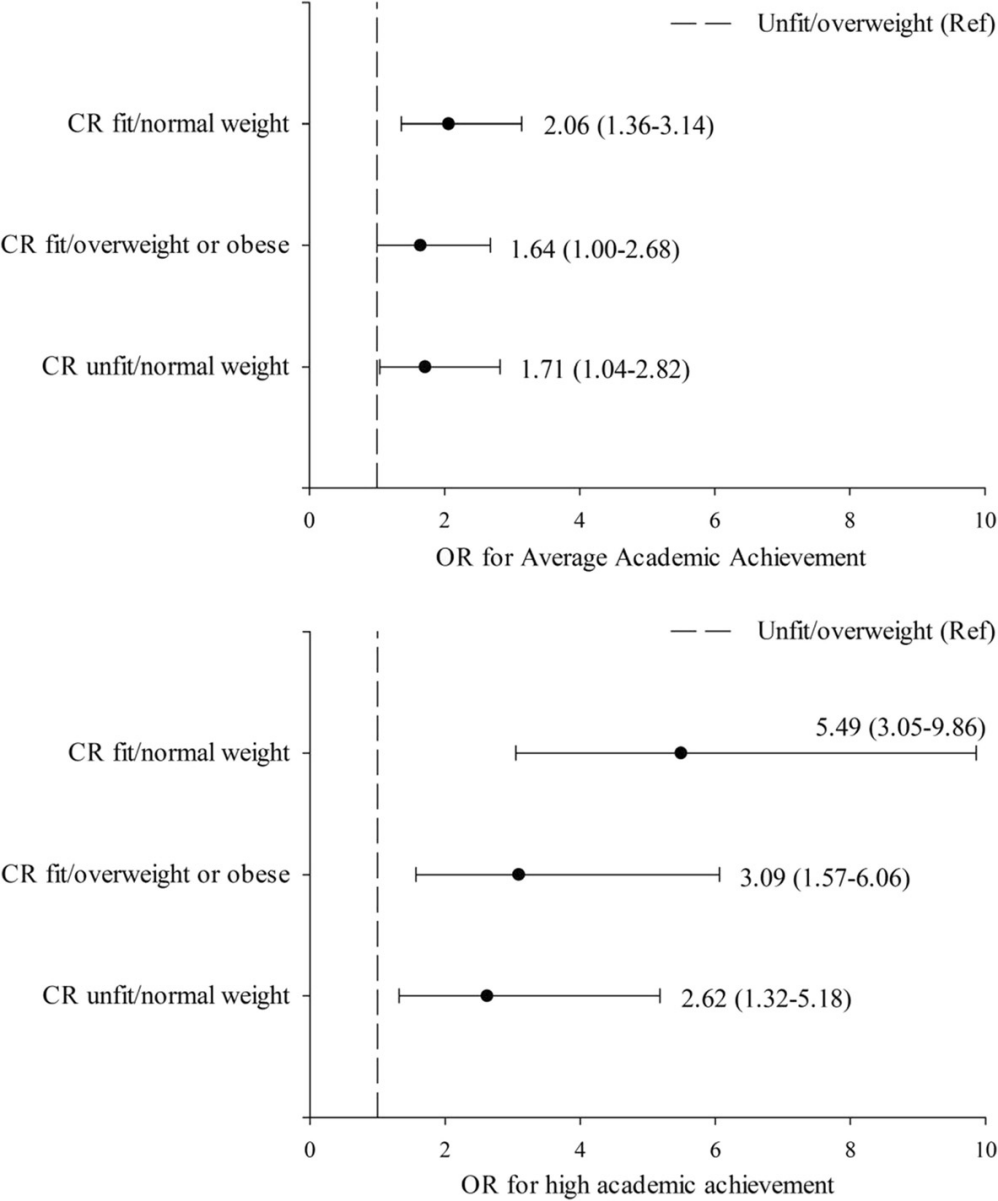
Odds ratio of predicting average and high academic achievement by cardiorespiratory fitness/weight status.

Figure 1 shows the OR of the relationship between academic achievement and the combined association of cardiorespiratory fitness/weight status. Students classified as cardiorespiratory fit/normal weight (OR = 5.49, 95% CI: 3.05-9.86, *p <* 0.001), as well as those classified as cardiorespiratory fit/overweight or obese (OR = 3.09, 95% CI: 1.57-6.06, *p =* 0.001), and cardiorespiratory unfit/normal weight (OR = 2.62, 95% CI: 1.32.5.18, *p* = 0.006) were more likely to have better academic performance, compared with those cardiorespiratory unfit/overweight or obese students.

## Discussion

The main finding of the present study was that cardiorespiratory fitness and weight status were independently and combined related to academic achievement in seventh-grade students independent of the different cohorts, providing further support that aerobically fit and lean students are more likely to have better performance at school regardless of the year that they were born.

Although several studies focusing on general physical fitness have established a relationship between academic performance and fitness or physical activity [33-35], we investigated a specific component of physical fitness. The results confirm and extend prior findings relating cardiorespiratory fitness with academic achievement in elementary school, independently of the birth year [9,12,21,36,37].

In a study among school children, Van Dusen et al. [10] have found that, among several physical fitness tests, cardiorespiratory fitness was the strongest fitness component related to academic achievement, which corroborated previous investigations [8,9]. More recently, two other studies with similar aims to the present study, from different countries, have also found that cardiorespiratory fitness was a predictor of academic achievement [22,26]. It has been shown that fit students were less likely to miss school and to do poorly on standardized tests [24,38], which are important risk factors often linked to dropping out of school.

The importance of this particular fitness component on academic achievement was seen in our study in the unadjusted and adjusted analysis, which means that individually or after adjustment for important covariates this variable is a predictor of academic achievement. Fit students had a 127% increased chance of reaching high academic achievement than cardiorespiratory unfit. These results demonstrate the important contribution of fitness, particularly cardiorespiratory capacity in students’ cognition; contributing to a growing body of literature about the relationship between physical fitness and academic achievement and supporting that a particular component of fitness is associated with general high academic performance. The findings of the present study are strengthened by the fact that three different cohorts were considered. This is to say that although they were all students from the 7^th^ grade, the sample consisted of students who were born three years apart, demonstrating the consistency of the results with different subsamples.

Causal inferences or explanations for the physical fitness-academic achievement association cannot be made from this study. However, mechanisms (i.e., physiological, psychological and behavioural) have been proposed to explain the link between fitness and academic performance. Studies have demonstrated that physical activity and fitness: 1) stimulate neural development, increasing the density of neuronal synapses [39], 2) increase levels of norepinephrine and endorphins, which are important to reduce stress and improve mood [40]; and 3) might increase the vasculature in the cerebral cortex [41]. At a biochemical level, physical activity and exercise augment the synthesis of brain-derived neurotrophic factor, also known as BDNF, which enhances brain plasticity by changing the structure of the neuron and strengthening its signalling capability [42,43]. Psychologically, physical fitness is positively associated with particular cognitive functioning related to attention and working memory [14,17]. Moreover, physical fitness contributes to accelerated psychomotor development, reduces anxiety and stress, and increases self-esteem, which are related to academic achievement [44]. Besides these suggested physiological and psychological effects, physical activity and physical fitness improve students’ behaviour in the learning context, consequently increasing the odds of better concentration and achievement [35].

Similarly to cardiorespiratory fitness, having normal weight was also associated with high academic achievement. Overweight students were 2.22 times and normal weight students were 3.72 times as likely to have high academic achievement as obese students. The relationship between weight status and academic achievement is congruent with some studies [8,23,24], but departs from other studies on which a relationship was not identified [9,10,12,20,21]. These disruptive findings perhaps are due to differences in BMI was quantified. We used a BMI classification; however in other studies BMI is treated as a continuous variable to prevent loss of information [22]. Further exploration research is required for the understanding of the relationship between academic achievement and weight status.

Although the relationship between academic achievement and weight status is not yet clearly established, two potential pathways have been proposed to associate weight status and academic outcomes. First, psychosocial pathways consider that overweight or obese students have lower self-esteem and body image [45], resulting in internalizing and externalizing behaviour problems that can affect students’ performance at school [25]. Second, physiological pathways consider that overweight and obesity are linked with health problems, leading to missed classes or lateness [46], both of which detrimentally affect school performance.

When analysing the combined association of cardiorespiratory fitness and weight status it was observed that for cardiorespiratory fit and normal weight students the odds of reaching high academic achievement increased by 449%. This result reinforced the finding for cardiorespiratory fitness and weight status and suggests that the combination of these two fitness components is a strong predictor of high academic achievement.

Because most children and adolescents are not physically fit [47], and fail to meet physical activity recommendation [48], school-based physical activity is important to offer or increase opportunities for physical activity [49]. Physical activity can be included in the school context in a number of ways without detracting from academic performance [34,49]. Considering that physical activity and physical fitness are related with academic achievement [12,21,37], schools should strive to meet recommendation of daily physical activity and offer students a balanced academic program that includes opportunities for a variety of daily physical activities.

In spite of the contribution of this study to the current understanding of the cardiorespiratory fitness- and weight status-academic achievement relationship, the study is not without some limitations. The cross-sectional design demonstrated an association between the variables, but did not indicate causality. Results need to be interpreted with some caution, because it is possible that the relationship between academic achievement and cardiorespiratory fitness was mediated by variable(s) not included in this study, such as socioeconomic status, parents’ education, and home background. Moreover, the used of categorical scholastic achievement instead of standardized academic outcomes could also be a limitation, due the fact that students marks may be related with other factors besides their cognitive performance.

## Conclusion and recommendations

The present study provides information regarding the independent influence of cardiorespiratory fitness and weight status on academic achievement in seventh-grade students. These findings suggest a synergic effect of cardiorespiratory fitness and weight status on academic achievement. Although other investigations have observed associations between cardiorespiratory fitness and weight status with academic achievement [22,26], the present research innovates by including three different cohorts, enabling to extend previous findings to children that were born in different years. These reinforce that children and adolescents should have more physical activity opportunities that allow improving cardiorespiratory fitness and weight status during school time.

## Competing interests

The authors declare that they have no competing interests.

## Authors’ contributions

Conception of the idea for the study: LBS. Development of the protocol, organization and funding: LBS, CM, AP, and SM. Responsible for the data analysis: AM. Supervision of the data analysis: SM and AP. Writing of the manuscript: AM and LBS. All authors have read the draft critically, to make contributions, and have approved the final text.

## Pre-publication history

The pre-publication history for this paper can be accessed here:

http://www.biomedcentral.com/1471-2431/14/176/prepub
